# PERCEPTIONS OF DIABETES SELF-MANAGEMENT AMONG MIDDLE AGED ADULTS

**DOI:** 10.1093/geroni/igae098.3685

**Published:** 2024-12-31

**Authors:** Willencia Louis-Charles, Hye Soo Lee, Wendy Rogers

**Affiliations:** University of Illinois Urbana-Champaign, Champaign, Illinois, United States; University of Illinois Urbana-Champaign, Champaign, Illinois, United States; University of Illinois Urbana-Champaign, Champaign, Illinois, United States

## Abstract

Older adults with diabetes are at higher risk of heart and kidney failure that arise due to mismanagement of the condition. Understanding the factors that influence self-management among middle-aged adults may enable healthcare providers to intervene before complications arise. We investigated whether knowledge, access to healthcare, illness identity, and diabetes distress emerged as factors that influence self-management for 11 middle-aged adults (aged 52-63, M=59.77, SD=5.17). All participants had type 2 diabetes. These participants completed questionnaires and a semi-structured interview either in-person or through Zoom during a one-time 2-hour session. Questionnaires included the Diabetes Knowledge Questionnaire (DKQ), Barriers to Access to Care Evaluation (BACE), Illness Identity Questionnaire (IIQ), and Diabetes Distress Scale (DDS). Following the questionnaires, they then were asked to elaborate on their experiences related to those themes and topics. Participants felt that they lacked knowledge, even though their scores on the DKQ were above 12, which is considered as satisfactory in the literature. Similarly, their BACE scores reflected enough access to healthcare, but many felt that they were not getting enough assistance. The IIQ data revealed that many participants felt more enrichment and rejection than engulfment and acceptance in reference to their diabetes. Many participants acknowledged feelings of distress that were not captured in their DDS. The mixed methods approach revealed that the standard measures are not diagnostic of the experiences, evidenced by the interview data. More in-depth evaluations of factors that affect health self-management for this population will be valuable to guide interventions before health complications arise.

